# Supplementary material for: A dream EEG and mentation database
Source: Nat Commun. 2025 Aug 13;16:7495. doi: 10.1038/s41467-025-61945-1 (PMC12350935; doi:10.1038/s41467-025-61945-1)
Supplement: Supplementary file 1 — Supplementary Information [file 41467_2025_61945_MOESM1_ESM.pdf]

Supplementary Information for:
DREAM: A Dream EEG and Mentation database

William Wong [1T*], Rubén Herzog [2T*], Kátia Cristine Andrade [3], Thomas Andrillon [2,4*], Draulio Barros de Araujo [3], Isabelle Arnulf [2], Somayeh Ataei [5,6,7], Giulia Avvenuti [8], Benjamin Baird [9], Michele Bellesi [10,11], Damiana Bergamo [8], Giulio Bernardi [8], Mark Blagrove [12], Nicolas Decat [2*], Çağatay Demirel [6], Martin Dresler [7], Jean-Baptiste Eichenlaub [13], Valentina Elce [8], Steffen Gais [14], Luigi De Gennaro [15], Jarrod Gott [7], Chihiro Hiramatsu [16], Bjørn Erik Juel [17,18], Karen R. Konkoly [19], Deniz Kumral [20], Célia Lacaux [2], Joshua J. LaRocque [21], Bigna Lenggenhager [22], Remington Mallett [19], Sérgio Arthuro Mota-Rolim [3], Yuki Motomura [16], Andre Sevenius Nilsen [18], Valdas Noreika [23*], Delphine Oudiette [2], Fernanda Palhano-Fontes [3], Jessica Palmieri [20], Ken A. Paller [19], Lampros Perogamvros [24], Antti Revonsuo [25,26], Elaine van Rijn [27], Serena Scarpelli [6], Monika Schönauer [20], Sarah F. Schoch [6,7,28], Francesca Siclari [29,30], Pilleriin Sikka [31,25,26,32], Johan Frederik Storm [18], Hiroshige Takeichi [33], Katja Valli [25,26*], Erin J. Wamsley [34], Jennifer M. Windt [4,35*], Jing Zhang [36], Jialin Zhao [6], Naotsugu Tsuchiya [1,37,*]

[T] These authors contributed equally.
* These authors jointly supervised this work
Corresponding author: naotsugu.tsuchiya@monash.edu

Affiliations:
1. School of Psychological Sciences and Turner Institute for Brain and Mental Health, Monash University, Clayton, Australia
2. Paris Brain Institute, Inserm-CNRS, Sorbonne Université, Paris, France
3. Brain Institute and Onofre Lopes University Hospital, Federal University of Rio Grande do Norte, Natal, Brazil
4. Monash Centre for Consciousness & Contemplative Studies, Monash University, Melbourne, Australia
5. Department of Neuropsychology, Faculty of Psychology, Ruhr University Bochum, Bochum, Germany
6. Donders Institute for Brain, Cognition and Behaviour, Nijmegen, Netherlands
7. Radboud University Medical Center, Donders Institute for Brain, Cognition and Behavior, Nijmegen, Netherlands
8. MoMiLab Research Unit, IMT School for Advanced Studies Lucca, Lucca, Italy
9. The University of Texas at Austin, Austin, USA
10. School of Physiology, Pharmacology and Neuroscience, University of Bristol, Bristol, UK
11. School of Biosciences and Veterinary Medicine, University of Camerino, Camerino (MC), Italy
12. Sleep Laboratory, School of Psychology, Swansea University, UK
13. Univ. Grenoble Alpes, Univ. Savoie Mont Blanc, CNRS, LPNC, Grenoble, France ; Institut Universitaire de France (IUF), Paris, France
14. Institute of Medical Psychology and Behavioral Neurobiology, University of Tübingen, Tübingen, Germany
15. Department of Psychology, University of Rome Sapienza, Rome, Italy
16. Faculty of Design, Kyushu University, Fukuoka, Japan
17. Institute of Basic Medical Sciences, University of Oslo, Oslo, Norway
18. Center for Sleep and Consciousness, University of Wisconsin–Madison, WI, USA
19. Department of Psychology and Cognitive Neuroscience Program, Northwestern University, Evanston, IL, USA
20. Institute of Psychology, Neuropsychology, University of Freiburg, Freiburg, Germany
21. Center for Neuroengineering and Therapeutics, University of Pennsylvania, Philadelphia, PA, USA
22. Association for independent research, Zurich, Switzerland
23. Centre for Brain and Behaviour, Department of Psychology, School of Biological and Behavioural Sciences, Queen Mary University of London, London, UK
24. University of Geneva, Geneva, Switzerland
25. Department of Psychology and Speech-Language Pathology, and Turku Brain and Mind Center, University of Turku, Finland
26. Department of Cognitive Neuroscience and Philosophy, University of Skövde, Sweden
27. Department of Psychology, Swansea University, Swansea, UK
28. Center of Competence Sleep & Health Zurich, University of Zurich, Zurich, Switzerland
29. Netherlands Institute for Neuroscience, Amsterdam, Netherlands
30. University of Lausanne, Switzerland
31. Department of Anesthesiology, Perioperative and Pain Medicine, School of Medicine, Stanford University, Stanford, USA

32. Department of Psychology, Stanford University, Stanford, USA
33. RIKEN Information R&D and Strategy Headquarters (R-IH), Japan
34. Department of Psychology and Program in Neuroscience, Furman University, Greenville, SC, USA
35. Department of Philosophy, Monash University, Clayton, Australia
36. Department of Cognitive Sciences, University of California, Irvine, CA, USA
37. Laboratory of Qualia Structure, ATR Computational Neuroscience Laboratories, Kyoto, Japan

Supplementary Table 1

**Supplementary Table 1.** Data fields of dataset records tables.		
**Field name**	**Description**	**Data type**
Filename	Filename of this sample’s PSG including the directory path relative to the /Data/PSG directory; does not include the leading slash; does include the file extension	String
Case ID	Unique ID of this sample within the dataset	String
Subject ID	Unique ID of this sample’s subject; it should match the patient code subfield of the “local patient identification” field of the sample EDF header	String
Experience	The dream experience reported for this sample (key: 2=experience, 1=experience without recall, 0=no experience, -1=experience with or without recall, -2=no experience or without recall, -3=experience with recall or no experience, -4=unknown)	Category
Treatment group	Unique ID of this sample’s experimental condition or treatment; documented in the “ExperimentalDescription.txt” file of the dataset if used	String
Duration	The duration of the PSG in seconds	Float
EEG sample rate	The sampling rate of the EEG in Hertz	Float
Number of EEG channels	The number of EEG signals in this sample	Integer

Last sleep stage	The scored sleep stage of the final epoch in the sample (key: 0=Wake, 1=N1/NREM1, 2=N2/NREM2, 3=N3/NREM3/NREM4, 5=R/REM)	Category
Has EOG	Whether EOG is included in the PSG (key: 0=no, 1=yes)	Boolean
Has EMG	Whether EMG is included in the PSG (key: 0=no, 1=yes)	Boolean
Has ECG	Whether ECG is included in the PSG (key: 0=no, 1=yes)	Boolean
Proportion artifacts	The proportion of signal data in the EEG that contain obvious artifacts; not necessarily precise	Float
Time of awakening	Time when this sample's PSG ends; leave blank if not known to within 3 hours' precision	Time
Participant age	Age of this sample's participant in whole years	Integer
Participant sex	Sex of this sample's participant (key: 0=male, 1=female, 2=other)	Category
Participant healthy	Whether the sample's participant is from a nominally relatively healthy population (key: 0=no, 1=yes)	Boolean
Has more data	Whether more detailed data than that contained in "Records.csv" is included in the dataset for this sample (key: 0=no, 1=yes)	Boolean
Remarks	Optional field for remarks	String

Supplementary Table 2

Supplementary Table 2. Data fields of the database "Data records" table.

**Field name**	**Description**	**Data type**

Key ID	Unique key for this record	Integer
Set ID	ID of dataset that this sample belongs to	Integer
Amendment	Amendment number of dataset that this sample belongs to	Integer
EDF filename	Filename of this sample’s PSG	String
Case ID	Unique ID of this sample within the dataset	String
Subject ID	Unique ID of this sample’s subject	String
Experience	The dream experience reported for this sample (experience, without recall, no experience, with or without recall, no experience or without recall, experience with recall or no experience, unknown)	Category
Treatment group	Unique ID of this sample’s experimental condition or treatment	String
Duration	The duration of the PSG in seconds	Float
EEG sample rate	The sampling rate of the EEG in Hertz	Float
Number of EEG channels	The number of EEG channels in this sample	Integer
Last sleep stage	The scored sleep stage of the final epoch in the sample (N1, N2, N3/NREM3/NREM4, REM, W)	Category
Has EOG	Whether EOG is included in the PSG	Boolean
Has EMG	Whether EMG is included in the PSG	Boolean

Has ECG	Whether ECG is included in the PSG	Boolean
Proportion artifacts	The proportion of signal data in the EEG that contain obvious artifacts	Float
Time of awakening	Time when this sample's PSG ends	Time
Subject age	Age of this sample's subject	Integer
Subject sex	Sex of this sample's participant (male, female, other)	Category
Subject healthy	Whether the sample's participant is from a relatively healthy population	Boolean

Supplementary Table 3

Table S3. Data fields of the database "Datasets" table.

**Field name**	**Description**	**Data type**
Key ID	Unique key for this record	Integer
Set ID	Unique ID for this dataset	Integer
Amendment	The amendment number for the dataset with given Set ID	Integer
Date entered	Date and time when this record was entered	Unix time
Common name	Short name of the dataset	String
Full name	Long name of the dataset	String

Corresponding contributor ID	The Key ID of a contributor from the “People” table for correspondence regarding this dataset	Integer
Data URL	The URL to access the dataset	URL
Contributors	Ordered list of all contributors responsible for the dataset; comma-separated	String
Number of samples	The number of experience reports in this dataset	Integer
Number of participants	The number of unique participants in this dataset	Integer
Proportion REM	The proportion of samples that end in a REM epoch	Float
Proportion N1	The proportion of samples that end in an N1/NREM1 epoch	Float
Proportion N2	The proportion of samples that end in an N2/NREM2 epoch	Float
Proportion W	The proportion of samples that end in a W epoch	Float
Proportion experience	The proportion of samples that had recalled experiences	Float
Proportion no-experience	The proportion of samples that had no experiences	Float
Proportion healthy	The proportion of samples that come from healthy participants	Float

Provoked awakening	Whether the study protocol instated provoked awakenings (yes, no, some)	Category
Time of awakening	General time of day when the awakenings occurred (morning, day, evening, night, mixed)	Category
Form of response	The protocol form of response given by participants to infer their experience (free, structured, categorical, other)	Category
Experimental description	A user description of the experiment conducted to collect these data (including any file decoding instructions, any treatment group codes, any categorization procedures, data acquisition details and preprocessing)	String
Study location	Name of location associated with the experiment conducted to collect these data	String
Study year	The year, or span of years, associated with the experiment conducted to collect these data	String
Publications	List of references to publications that have used this dataset	String
Correspondence note	A user note of correspondence details	String
Data restriction note	A user note of data restriction details, data request instructions, data sharing agreements, and time frame of response	String
Date approved	Date and time when this record was approved by the curator; set as N/A if not approved	Unix time

Accessibility	Level of accessibility of the dataset (open, conditional, private, other)	Category
Revoked	Whether this entry has been revoked for inclusion in the collection	Boolean
Latest amendment	Whether this entry is the latest amendment of all datasets with the same Set ID	Boolean

Supplementary Methods 1—Data quality assessment

The PSG recordings of the DREAM database are stored in European Data Format (EDF). The conversion of raw EEG signals to EDF can sometimes deteriorate the quality of the data. Two issues can arise during the conversion: signal clipping and decreased resolution. The occurrence of these issues depends on the range of accepted values that is established prior to the EDF conversion. If the range of accepted values is too narrow with respect to the actual physical values, the signal can be clipped. Conversely, if the accepted values too largely exceed the physical signal, a decrease in resolution is observed.

To verify the presence of signal clipping and decreased resolution in the uploaded data, we carried out a data quality assessment on each PSG using *checkEDF*, a semi-automatic MATLAB toolbox we developed. To check for signal clipping, the toolbox flagged channels whose amplitude distribution lies between +/-500 µV and peaks at a certain value within that range. To check for low resolution, it flagged channels which presented a minimal difference in amplitude between adjacent points of >0.2 µV. The toolbox returned for each channel a visualization of the amplitude range and step, which we then visually inspected. Recordings containing such problematic channels were re-exported with

threshold amplitude values at +/-1000 µV, which allows for a preserved data resolution while preventing any signal clipping.

Supplementary Methods 2—Data use demonstration: additional methods and results

Behavioral analyses: contingency table

All pairwise comparisons of counts in the contingency table (Supplementary Table 4)—within each row and within each column—were significantly different (Fisher's exact test, $p_{adj} < 0.05$; correction for multiple comparisons using Hochberg's step-up procedure, $m = 30$) except for all "N1"-"REM" and "N2"-"N3" comparisons (Fisher's exact test, $p_{adj} = 0.71$) and the "N1"-"N3" comparison within the "Experience without recall" column (Fisher's exact test, $p_{adj} = 0.058$).

Supplementary Table 4

Sleep stage vs. Experience contingency table's deviance from the assumption of independence. See also Table 4 from the main text.

	**No experience**	**Experience without recall**	**Experience**
**N1**	-61%	-84%	+33%
**N2**	+28%	+39%	-15%
**N3/NREM3/NREM4**	+45%	+93%	-27%
**REM**	-40%	-59%	+22%

Behavioral analyses: Generalized linear mixed-effects model

We performed generalized linear mixed-effects (GLME) modeling with Markov chain Monte Carlo techniques on the same data using the MCMCglmm R package, taking *Experience* as the outcome variable, *Last sleep stage* as the fixed effect, and *Subject ID* and study (*Set ID*) as random effects. The model had the following formula in Wilkinson notation:

$$Experience \sim LastSleepStage + (SetID) + (SubjectID) - 1$$

Experience was a categorical variable modeled as two latent variables: $L_{EWR:NE}$ and $L_{E:NE}$, as the log-odds of their respective "Experience" categories against *NE* as the reference category (e.g., $L_{EWR:NE}$ is the log-odds for "Experience without recall" against "No experience"). *Last sleep stage*, as the categorical fixed effect, was expanded using the dummy coding system to four dummy variables—one for each sleep stage—and expanded again for each additional latent outcome variable. Here, we denote their coefficients as $F_{N1,EWR:NE}$, $F_{N1,E:NE}$, $F_{N2,EWR:NE}$, etc. To decide the covariance matrix structure to model for the random effects, a total of four GLME models were tested where each random component had either an isotropic diagonal variance or an anisotropic one, and the model with the lowest deviance information criterion was selected. This turned out to be the model where the *Set ID*'s was isotropic and the *Subject ID*'s was anisotropic.

The selected model was fit by estimating the joint posterior distribution of the modeled variables using moderately diffuse priors, and a fixed residual variance of unity. We found that a stronger prior distribution for the random effect covariance matrix was necessary for convergence of the model; we used an inverse Wishart distribution with scale parameter $\mathbf{\Psi} = 1$ and $\nu = 5$ degrees of freedom. One million MCMC iterations were computed and thinned to produce 45,000 samples for the posterior distribution.

Supplementary Table 5 gives the estimated fixed effects coefficients. The *Set ID* random effect contributed a maximum *a posteriori* standard deviation of 2.27 logits, and the *Subject ID* random effect contributed 1.04 and 4.78 logits to the *EWR* and *E* latent outcome variables respectively.

Supplementary Table 5

Estimated fixed effect coefficients of the logistic GLME model. Their central tendencies (i.e., maximum *a posteriori*) and 95% credible intervals (CI) are given; and posteriors whose CI do not include zero are marked with asterisks (two-tailed, not adjusting for multiple comparisons). Also given in the three rightmost columns are the central tendencies of the *Experience* posterior distributions decoded, given fixed effects.

Outcome variable	Fixed effect	Maximum a posteriori estimate	Lower 95% CI	Upper 95% CI	P(Experience = NE)	P(Experience = EWR)	P(Experience = E)
$L_{EWR:NE}$	F_{N1}	-5.16*	-9.25	-1.52	.020	<.001	.979
$L_{E:NE}$		3.87*	2.05	8.72			
$L_{EWR:NE}$	F_{N2}	-3.48*	-5.56	-1.73	.203	.006	.791
$L_{E:NE}$		1.36	-0.01	3.47			
$L_{EWR:NE}$	F_{N3}	-2.48	-5.26	0.16	.685	.058	.257
$L_{E:NE}$		-0.98	-3.92	0.96			
$L_{EWR:NE}$	F_{REM}	-3.68*	-6.08	-1.89	.072	.002	.926
$L_{E:NE}$		2.55*	1.07	5.76			

The expected qualitative trend of deeper NREM sleep stages associating with lower odds of reports of "Experience with recall" of content against "No experience" reports is again supported by this result. More than a 0.999 fraction of the sampled posterior density lies in the region where $F_{N1,E:NE} > F_{N2,E:NE} > F_{N3,E:NE}$. The same trend is found for the odds of "Experience with recall" of content against "Experience without recall", quantified by derived latent variable $L_{E:EWR} = L_{E:NE} – L_{EWR:NE}$. A 0.996 fraction of the posterior lies in the region where $F_{N1,E:EWR} > F_{N2,E:EWR} > F_{N3,E:EWR}$. There is not a clear trend for the odds of "Experiences without recall" of content against "No experiences". A 0.651 fraction of the posterior lies in the region where $F_{N1,EWR:NE} < F_{N2,EWR:NE} < F_{N3,EWR:NE}$, and a 0.032 fraction for the reverse trend. The odds of "Experience" against "No experience" for the REM sleep stage graphically also appears higher than for N2 and lower than for N1. Indeed, a 0.992 fraction of the sampled posterior density lies in the region where $F_{N1,E:NE} > F_{REM,E:NE} > F_{N2,E:NE}$.

EEG analysis: Data preparation

EEG data recorded in the 10-20 system were referenced to the average of the two mastoids (with the exception of *Zhang* dataset, which was already referenced). For these datasets, a left and right EOG as well as chin EMG derivations were available. The *Siclari* dataset was recorded with a high-density net not in the 10-20 system. For this dataset, frontal (F4), central (C4) and occipital (O2) electrodes were approximated using electrodes E214, E183 and E149 respectively and according to the HydroCel-GSN-256-v10 layout provided by the manufacturer. These electrodes were referenced to E94 (left mastoid). The chin EMG was reconstructed by computing a bipolar derivation using two electrodes on the jaw (E251 and E233). Left and right EOG were also reconstructed by subtracting electrode E1 to E94 (left mastoid) for the right EOG and E54 to E190 (right mastoid) for the left EOG.

EEG analysis: Power spectral density

For each epoch prior to report, one central electrode (C3 or C4) was used to compute the s PSDs using the *mne* Python library with the multitaper method in the 0.4–35 Hz frequency range. PSDs were normalized by the total power, such that the total area under the curve adds up to one. All the PSDs corresponding to a specific sleep stage were averaged within a dataset, and then globally across six datasets.

EEG analysis: Automatic sleep scoring

We applied a previously-validated automatic sleep scoring algorithm[1] to the six datasets. The code for the algorithm and trained model can be found at https://github.com/Stanford-STAGES/stanford-stage. Each data segment was processed separately and contained at least 30s of data with two EEG channels (a central and occipital channel, whichever available in the dataset), two EOG channels (left and right) and one chin EMG channel. We extracted the hypnodensities computed by the algorithm (the probability of each AASM sleep stage) for two contiguous segments of 15-second corresponding thus to the last 30 seconds of data prior to a report, and averaged them. We took the sleep stage with the maximum probability as a summary output of the hypnodensities for the analysis in Figure 4. We used the five AASM stages (wakefulness, NREM stage 1 or N1, NREM stage 2 or N2, NREM stage 3 or N3, and REM sleep) for the analysis in Figure 3. We used a reduced sleep staging, where N1, N2 and N3 were merged into the NREM stage for Figure 4.

To measure the performance of the automatic sleep staging algorithm in Figure 3, we computed the "subset accuracy" between the stages obtained from human experts (H) and the stages obtained with the automatic algorithm (A) as follows:

$$Acc = \frac{1}{n}\sum_{i=1}^{n} I\left[H_i = A_i\right],$$

where n is the number of epochs, H_i and A_i is the sleep stage of the i-th epoch according to human and automatic scoring, respectively. $I[.]$ is the Iverson bracket function, which yields 1 if both classes are equal and 0 otherwise. We computed the accuracy for 1,000 random subsamples of 50% of the epochs, and the average accuracy across those subsamples was reported. We used this metric instead of Cohen's kappa because some datasets had only one sleep stage, which made the latter measure to be undefined.

We applied Bayesian ANOVA to the hypnodensities with the main effects of automatically-scored sleep stage (three levels, W, NREM and REM) and dream report (three levels, E, NE, EWR), and a random grouping effect of dataset (six levels, one for each dataset). The ANOVA was implemented in JASP 0.16.3.

EEG analysis: Feature extraction and experience classification

We used only the F4, C4 and O2 electrodes, as they were present in the 6 aforementioned dataset. EEG data was bandpass filtered in the 0.5-35 Hz range and then downsampled to 128 Hz (in cases where the sampling rates were higher) to avoid differences in the extracted features due to different sampling rates. PSD were estimated using the *mne* python library with the multitaper method in the 0.5-35 Hz range for all the datasets, epochs and channels. Normalized (by the total PSD power) PSDs were extracted for each epoch in 6 different frequency bands: delta (0.5-4 Hz), theta (4.1-8), alpha (8.1-11 Hz), sigma (11.1-15 Hz), beta (15.1-20 Hz) and gamma (20.1-35 Hz). In addition, the set of 22 features provided by the catch22 library[2] were estimated for each epoch and channel in the broadband range (bb, 0.5-35 Hz) and on each bandpass filtered (bf) version of the data using the aforementioned frequency ranges.

All the epochs associated with the same sleep stage were taken together across studies. For each sleep stage, a XGBoost[3] algorithm was trained to discriminate between “Experience” and “No

Experience". A 5-fold cross-validation procedure was used, where 4 folds were used for training and the remaining was used for testing. This procedure was repeated 5 times, using each possible fold as the test set and the rest 4 as the training set, and classifiers performance was obtained by averaging across the 5 test sets. The performance was quantified using the area under the receiver operating characteristic curve (AUC). To avoid idiosyncrasies related to a specific partition of the data, the 5-fold validation procedure was repeated 200 times for each comparison, leading to a distribution of AUC values. To evaluate the departure from chance performance, a null distribution was generated by repeating the same procedure but shuffling the labels for dream experience of the epochs. The empirical AUC distribution was compared against the null distribution via a Wilcoxon rank sums non-parametric test to evaluate the difference of medians. A Bernoulli correction for multiple comparisons was applied for all the p-values.

References

1. Stephansen, J. B. *et al.* Neural network analysis of sleep stages enables efficient diagnosis of narcolepsy. *Nat Commun* **9**, 5229 (2018).
2. Lubba, C. H. *et al.* catch22: CAnonical Time-series CHaracteristics. *Data Min Knowl Disc* **33**, 1821–1852 (2019).
3. Chen, T. & Guestrin, C. XGBoost: A Scalable Tree Boosting System. in *Proceedings of the 22nd ACM SIGKDD International Conference on Knowledge Discovery and Data Mining* 785–794 (2016). doi:10.1145/2939672.2939785.
